# Supplementary material for: The Genetic Markers of Knee Osteoarthritis in Women from Russia
Source: Biomedicines. 2024 Apr 2;12(4):782. doi: 10.3390/biomedicines12040782 (PMC11048526; doi:10.3390/biomedicines12040782)
Supplement: Supplementary file 1 [file biomedicines-12-00782-s001.zip › Table S1.pdf]

Table S1. VNTR allele frequencies of the *ACAN* gene polymorphism in women with knee OA and controls.

Group	Alleles											
	19	20	21	22	23	24	25	26	27	28	29	30
Knee OA (N=137)	0	1 (0,004)	1 (0,004)	2 (0,007)	2 (0,007)	0	6 (0,022)	36 (0,131)	141 (0,515)	78 (0,285)	5 (0,018)	2 (0,007)
Control (N=161)	1 (0,003)	0	1 (0,003)	0	1 (0,003)	1 (0,003)	13 (0,040)	52 (0,162)	128 (0,398)	117 (0,363)	8 (0,025)	0
Total (N=298)	1 (0,002)	1 (0,002)	2 (0,003)	2 (0,003)	3 (0,005)	1 (0,002)	19 (0,033)	88 (0,148)	269 (0,451)	195 (0,327)	13 (0,021)	2 (0,003)
