# Supplementary material for: The Genetic Markers of Knee Osteoarthritis in Women from Russia
Source: Biomedicines. 2024 Apr 2;12(4):782. doi: 10.3390/biomedicines12040782 (PMC11048526; doi:10.3390/biomedicines12040782)
Supplement: Supplementary file 1 [file biomedicines-12-00782-s001.zip › Table S2.pdf]

**Table S2. Allele and genotype frequencies of candidate genes in patients with knee OA and in control group**

| Groups | N | Allele frequencies | | | | Genotype frequencies | | | | | |
|---|---|---|---|---|---|---|---|---|---|---|---|
| rs6539153 (*CHST11*) | | | | | | | | | | | |
| Control | 161 | C | 134 (0,416) | T | 188 (0,584) | CC | 32 (0,199) | CT | 70 (0,435) | TT | 59 (0,366) |
| Knee OA | 123 | | 108 (0,439) | | 138 (0,561) | | 24 (0,195) | | 60 (0,488) | | 39 (0,317) |
| rs226794 (*ADAMTS5*) | | | | | | | | | | | |
| Control | 161 | G | 276 (0,857) | A | 46 (0,143) | GG | 116 (0,721) | GA | 44 (0,273) | AA | 1 (0,006) |
| Knee OA | 135 | | 222 (0,822) | | 48 (0,178) | | 90 (0,667) | | 42 (0,311) | | 3 (0,022) |
| rs2830585 (*ADAMTS5*) | | | | | | | | | | | |
| Control | 161 | C | 284 (0,882) | T | 38 (0,118) | CC | 126 (0,782) | CT | 32 (0,199) | TT | 3 (0,019) |
| Knee OA | 137 | | 237 (0,865) | | 37 (0,135) | | 103 (0,752) | | 31 (0,226) | | 3 (0,022) |
| rs1042667 (*SOX9*) | | | | | | | | | | | |
| Control | 162 | A | 188 (0,580) | C | 136 (0,420) | AA | 59 (0,364) | AC | 70 (0,432) | CC | 33 (0,204) |
| Knee OA | 137 | | 173 (0,631) | | 101 (0,369) | | 56 (0,409) | | 61 (0,445) | | 20 (0,146) |
| rs2229989 (*SOX9*) | | | | | | | | | | | |
| Control | 161 | C | 244 (0,758) | T | 78 (0,242) | CC | 92 (0,571) | CT | 60 (0,373) | TT | 9 (0,056) |
| Knee OA | 137 | | 206 (0,752) | | 68 (0,248) | | 78 (0,579) | | 50 (0,365) | | 9 (0,066) |
| rs7217932 (*SOX9*) | | | | | | | | | | | |
| Control | 161 | C | 168 (0,522) | T | 154 (0,478) | CC | 44 (0,273) | CT | 80 (0,497) | TT | 37 (0,230) |
| Knee OA | 124 | | 125 (0,504) | | 123 (0,496) | | 35 (0,282) | | 55 (0,444) | | 34 (0,274) |
| rs1107946 (*COL1A1*) | | | | | | | | | | | |
| Control | 161 | C | 249 (0,773) | A | 73 (0,227) | CC | 100 (0,621) | CA | 49 (0,304) | AA | 12 (0,075) |
| Knee OA | 139 | | 224 (0,806) | | 54 (0,194) | | 90 (0,647) | | 44 (0,317) | | 5 (0,036) |
| rs1800012 (*COL1A1*) | | | | | | | | | | | |
| Control | 161 | G | 275 (0,854) | T | 47 (0,146) | GG | 118 (0,733) | GT | 39 (0,242) | TT | 4 (0,025) |
| Knee OA | 139 | | 235 (0,845) | | 43 (0,155) | | 102 (0,734) | | 31 (0,223) | | 6 (0,043) |
