# Supplementary material for: The Genetic Markers of Knee Osteoarthritis in Women from Russia
Source: Biomedicines. 2024 Apr 2;12(4):782. doi: 10.3390/biomedicines12040782 (PMC11048526; doi:10.3390/biomedicines12040782)
Supplement: Supplementary file 1 [file biomedicines-12-00782-s001.zip › Table S3.pdf]

Table S3. Allele and genotype frequencies of GWAS-replication genes in patients with knee OA and in control group

Groups	N	Allele frequencies				Genotype frequencies					
Rs 1298744 (*DOT1L*)											
Control	162	C	211 (0,651)	G	113 (0,349)	CC	68 (0,420)	CG	75 (0,463)	GG	19 (0,117)
Knee OA	139		183 (0,658)		95 (0,342)		56 (0,403)		71 (0,511)		12 (0,086)
Rs2302061 (*DOT1L*)											
Control	161	C	41 (0,127)	G	281 (0,873)	CC	1 (0,006)	CG	39 (0,242)	GG	121 (0,752)
Knee OA	139		50 (0,180)		228 0,820		6 0,043		38 (0,273)		95 (0,683)
Rs3204689 (*ALDH1A2*)											
Control	161	C	203 (0,630)	G	119 (0,370)	CC	67 (0,416)	CG	69 (0,429)	GG	25 (0,155)
Knee OA	139		183 (0,658)		95 (0,342)		56 (0,403)		71 (0,511)		12 (0,086)
Rs6976 (*GLT8D1*)											
Control	161	C	170 (0,528)	T	152 (0,472)	CC	43 (0,267)	CT	84 (0,522)	TT	34 (0,211)
Knee OA	136		159 (0,585)		113 (0,415)		51 (0,375)		57 (0,419)		28 (0,206)
Rs11177 (*CNL3*)											
Control	161	C	162 (0,503)	T	160 (0,497)	CC	42 (0,261)	CT	78 (0,484)	TT	41 (0,255)
Knee OA	139		164 (0,590)		114 (0,410)		50 (0,360)		64 (0,460)		25 (0,180)
Rs4836732 (*ASTN2*)											
Control	161	T	170 (0,528)	C	152 (0,472)	TT	40 (0,248)	TC	90 (0,559)	CC	31 (0,193)
Knee OA	139		140 (0,504)		138 (0,496)		35 (0,251)		70 (0,504)		34 (0,245)
Rs9350591 (*FILIP/SENP6*)											
Control	161	C	285 (0,885)	T	37 (0,115)	CC	127 (0,789)	CT	31 (0,192)	TT	3 (0,019)
Knee OA	137		233 (0,850)		41 (0,150)		97 (0,708)		39 (0,285)		1 (0,007)
Rs6094710 (*NCOA3*)											
Control	161	A	15 (0,047)	G	307 (0,953)	AA	1 (0,006)	AG	13 (0,081)	GG	147 (0,913)
Knee OA	137		14 (0,051)		260 (0,949)		0		14 (0,102)		123 (0,898)
Rs11841874 (*MCF2L*)											
Control	161	A	283 (0,879)	G	39 (0,121)	AA	125 (0,776)	AG	33 (0,205)	GG	3 (0,019)
Knee OA	122		210 (0,861)		34 (0,139)		91 (0,745)		28 (0,230)		3 (0,025)
Rs7639618 (*DVWA*)											
Control	161	C	267 (0,829)	T	55 (0,171)	CC	115 (0,714)	CT	37 (0,230)	TT	9 (0,056)
Knee OA	139		211 (0,759)		67 (0,241)		81 (0,583)		49 (0,352)		9 (0,065)
Rs835787 (*CHST11*)											
Control	162	T	220 (0,679)	C	104 (0,321)	TT	72 (0,444)	CT	76 (0,470)	CC	14 (0,086)
Knee OA	115		160 (0,696)		70 (0,304)		56 (0,487)		48 (0,417)		11 (0,096)
